# Charakteristika von Plattenepithelkarzinomen auf Hidradenitis‐suppurativa‐Läsionen – eine Fallserie

**DOI:** 10.1111/ddg.15708_g

**Published:** 2025-07-14

**Authors:** Nessr Abu Rached, Riina Käpynen, Yannik Haven, Lennart Ocker, Carolin Frost, Eggert Stockfleth, Falk G. Bechara

**Affiliations:** ^1^ Klinik für Dermatologie Venerologie und Allergologie Internationales Zentrum für Hidradenitis suppurativa / Acne inversa (ICH) Ruhr‐Universität Bochum; ^2^ Klinik für Dermatologie Venerologie und Allergologie Hautkrebszentrum Ruhr‐Universität Bochum

**Keywords:** Acne inversa, HS, HPV, Hidradenitis suppurativa, Malignom, Plattenepithelkarzinom, acne inversa, HS, HPV, Hidradenitis suppurativa, malignoma, squamous cell carcinoma

Sehr geehrte Herausgeber,

Hidradenitis suppurativa (HS) ist eine chronisch‐entzündliche Hauterkrankung, die hauptsächlich die intertriginösen Regionen betrifft. Chronische Entzündung, virale Induktion durch humane Papillomaviren (HPV), chronische Wundheilungsstörungen und hoher Tabakkonsum erhöhen das Risiko für die Entwicklung eines kutanen Plattenepithelkarzinoms (cSCC).^1^ Insgesamt ist cSCC bei HS‐Läsionen eine seltene, aber sehr schwerwiegende Komplikation der HS (Abbildung 1). Daher haben wir unsere Fälle mit cSCC retrospektiv gesammelt, um charakteristische Merkmale zu identifizieren.

**ABBILDUNG 1:**
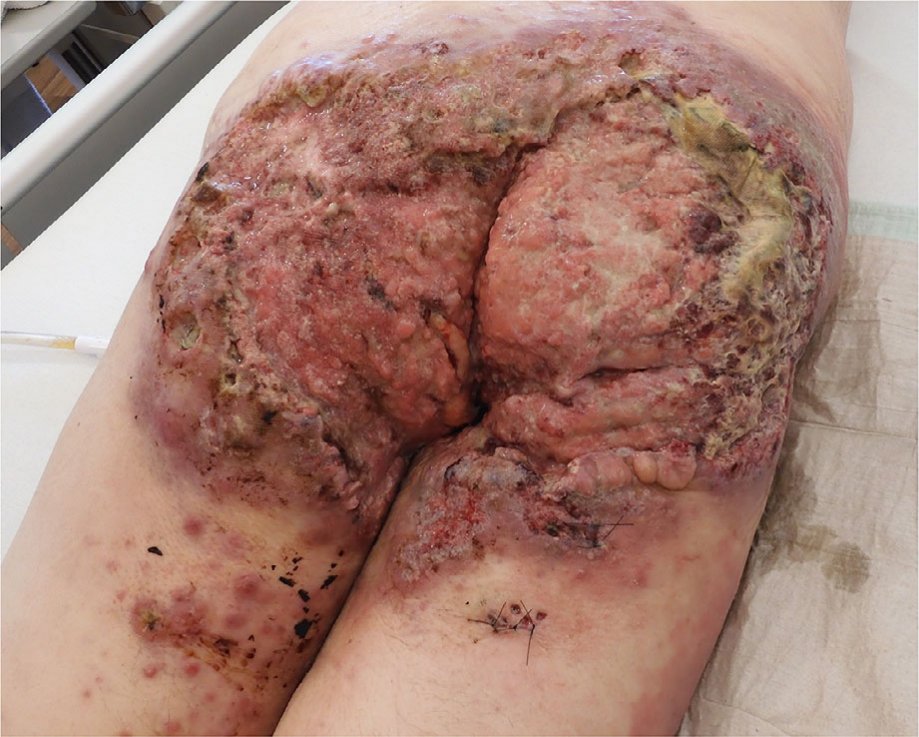
Klinisches Bild eines Plattenepithelkarzinoms auf Hidradenitis‐suppurativa‐Läsionen: Es handelt sich um ein ulzeriertes Plattenepithelkarzinom, das die gesamte Gesäß‐ und Perianalregion befällt.

Wir führten eine retrospektive Analyse an einer Kohorte von sieben Patienten mit HS durch, bei denen ein kutanes Plattenepithelkarzinom diagnostiziert wurde. Die Kohorte bestand überwiegend aus Männern (85,7 %) mit einem medianen Alter von 60 Jahren bei Erstdiagnose des kutanen Plattenepithelkarzinoms (Bereich: 42–65 Jahre) (Tabelle 1). Die mediane Erkrankungsdauer der HS zum Zeitpunkt der Diagnose des kutanen Plattenepithelkarzinoms betrug 28 Jahre (Bereich: 14–47 Jahre), was die anhaltende entzündliche Belastung im Zusammenhang mit der malignen Transformation bei diesen Patienten verdeutlicht. Alle Patienten wurden als Stadium III nach Hurley klassifiziert. Der mediane BMI betrug 25,7 kg/m^2^ (Bereich: 21–36,4 kg/m^2^), was für eine typische HS‐Kohorte niedrig ist. Der Raucherstatus ergab 42,9 % aktive Raucher und 57,1 % Ex‐Raucher mit einer medianen Pack‐Year‐Anzahl von 30 (Spanne: 0–45). Die häufigsten Lokalisationen von HS waren die perineale/perianale Region (100 %), die gluteale Region (85,7 %) und die inguinale Region (71,4 %). Bei 42,9 % der Patienten wurde ein HS‐assoziiertes Lymphödem diagnostiziert und 57,1 % hatten eine Biologikatherapie erhalten, darunter Secukinumab (14,3 %) und Adalimumab (42,9 %).

**TABELLE 1:** Persönliche, tumorspezifische, HS‐spezifische und laborchemische Merkmale von Patienten mit Plattenepithelkarzinom auf Hidradenitis‐suppurativa‐Läsionen (n  =  7).

Parameter	Wert
***Persönliche und HS‐spezifische Merkmale***	
Geschlecht, n (%)	
Weiblich	1 (14,3)
Männlich	6 (85,7)
Alter bei Diagnose vom cSCC, Median (Bereich), Jahre	60 (42–65)
Alter bei Beginn der HS, Median (Bereich), Jahre	29,3 (14–51)
HS‐Erkrankungsdauer bei Diagnose vom cSCC, Median (Bereich), Jahre	28 (14–47)
BMI, Median (Bereich), kg/m^2^	25,7 (21–36,4)
Familienanamnese in Bezug auf HS, n (%)	
Positiv	2 (28,6)
Negativ	5 (71,4)
Raucherstatus, n (%)	
Aktiver Raucher	3 (42,9)^*^
Nicht Raucher	0 (0)
Ehemaliger Raucher	4 (57,1)
Raucherjahre [pack‐years], Median (Bereich)	30 (0–45)^*^
Hurley Stadium, n ( %)	
Hurley I	0 (0)
Hurley II	0 (0)
Hurley III	7 (100)
Axilläre Beteiligung der HS, n (%)	4 (57,1)
Brustbeteiligung der HS, n (%)	1 (14,3)
Genitale Beteiligung der HS, n (%)	4 (57,1)
Gluteale Beteiligung der HS, n (%)	6 (85,7)
Inguinale Beteiligung der HS, n (%)	5 (71,4)
Perineale/perianale Beteiligung der HS, n (%)	7 (100)
HS‐assoziiertes Lymphödem, n (%)	3 (42,9)
Biologische Vorbehandlung, n (%)	4 (57,1)
Secukinumab Vorbehandlung, n (%)	1 (14,3)
Adalimumab Vorbehandlung, n (%)	3 (42,9)
Anzahl der betroffenen HS‐Stellen, Median (Bereich)	4 (3–9)
***Laborparameter zum Zeitpunkt der Diagnose vom cSCC***	
CRP in mg/l, Median (Bereich)	124,4 (79,9–186,6)
Leukozyten Zellen/µl, Median (Bereich)	12 010 (8850–41 130)
Thrombozyten Zellen/µl, Median (Bereich)	398 000 (325 000–565 000)
***Tumorspezifische Daten***	
Anzahl der cSCC‐spezifischen Todesfälle, n (%)	4 (57,1)
Zeit bis zum Tod in Monaten, Median (Bereich)	3 (0–34)
Lokalisation des cSCC, n (%)	
Perianal	2 (28,6)
Gluteal	3 (42,9)
Oberschenkel	1 (14,3)
Sakral	1 (14,3)
Tumordifferenzierung	
Grad 1, n ( %)	4 (57,1)
Grad 2, n ( %)	2 (28,6)
Grad 3, n ( %)	1 (14,3)
Anzahl der Patienten mit Metastasen, n ( %)	3 (42,9)

*Abk*.: HS, Hidradenitis suppurativa; cSCC, kutanes Plattenepithelkarzinom

* Ein HS‐Patient mit 60 *joint‐years* ohne Tabakkonsum.

Hinsichtlich der tumorspezifischen Daten starben 57,1 % der Patienten an cSCC mit einer mittleren Zeit bis zum Tod von 3 Monaten (Bereich: 0–34 Monate). Die häufigsten Lokalisationen des cSCC waren die Glutealregion (42,9 %) und die Perianalregion (28,6 %). Die Tumordifferenzierungsgrade waren Grad 1 (57,1 %), Grad 2 (28,6 %) und Grad 3 (14,3 %). Metastasen waren bei 42,9 % der Patienten vorhanden. Ein Patient hatte kutane, lymphogene und pulmonale Metastasen, ein Patient hatte lymphogene Metastasen und ein Patient hatte Knochenmetastasen. Die anderen vier Patienten hatten keine lokalen oder Fernmetastasen des cSCC.

In Übereinstimmung mit der Literatur bestätigte unsere Gruppe, dass das cSCC bei HS‐Läsionen hauptsächlich Männer und die untere Körperregion (insbesondere perianal/perineal) betrifft. HS‐Patienten mit einem hohen Krankheitsschweregrad, der sich in hohen Entzündungsmarkern und einem Hurley‐III‐Befund widerspiegelt, scheinen besonders häufig von cSCC betroffen zu sein. Bemerkenswert ist der niedrige mediane BMI unserer HS‐Kohorte von 25,7 kg/m^2^ (Bereich: 21–36,4 kg/m^2^). Der typische mittlere BMI unserer HS‐Kohorte beträgt 31,5 kg/m^2^ (Standardabweichung ± 6,7 kg/m^2^).^3^ Ein tumorbedingter Gewichtsverlust könnte den großen Unterschied erklären. In den zuvor veröffentlichten Fallstudien wurde der BMI nicht angegeben. Obwohl der Grund für den Gewichtsverlust spekulativ bleibt, ist er wahrscheinlich die Hauptursache.

Im Allgemeinen sind cSCC in HS‐Läsionen aggressiver als cSCC in anderen Lokalisationen. Dies spiegelt sich in der hohen Mortalitätsrate von 57,1 % mit einer mittleren Zeit bis zum Tod von 3 Monaten (Bereich: 0–34 Monate) wider. Es ist jedoch wichtig zu betonen, dass es sich bei zwei von drei noch lebenden Patienten um neu diagnostizierte Fälle von cSCC handelt, so dass die Sterblichkeitsrate noch höher sein könnte. Ein entzündliches Tumormikromilieu könnte eine wichtige Rolle bei der Erklärung der hohen Aggressivität von cSCC bei HS spielen. Die entzündliche Mikroumgebung der HS mit den Zytokinen Tumornekrosefaktor (TNF)‐α, Interleukin (IL)‐6, IL‐17 und IL‐1β könnte die Tumorentwicklung begünstigen. Aber auch der langjährige Tabakkonsum sollte bei der Tumorentstehung nicht unterschätzt werden. Auffällig ist, dass alle unsere Patienten lange geraucht hatten (Median der pack‐years: 30 Jahre). Auch bestimmte Inhaltsstoffe von Zigaretten und Cannabinoide können die DNA schädigen und die Tumorentstehung fördern.^4^, ^5^ Ein weiterer Grund für das schlechte Outcome von HS‐Patienten mit cSCC könnte die Herausforderung der histologischen Diagnose von cSCC bei HS sein. Die massive Entzündung kann es dem Pathologen erschweren, die Diagnose cSCC zu stellen (Abbildung 2). Die hohen CRP‐Werte hängen wahrscheinlich mit der schweren Entzündung bei HS zusammen. Eine andere Möglichkeit ist, dass die hohen CRP‐Spiegel zum Teil durch den Tumor verursacht werden. Beide Hypothesen sind möglich und können durch die Fallserie nicht verifiziert werden.

**ABBILDUNG 2:**
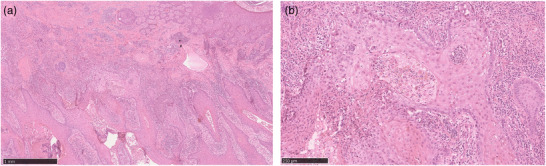
Die Abbildung zeigt ein Plattenepithelkarzinom auf Hidradenitis‐suppurativa‐Läsionen in Hämatoxylin‐Eosin (HE)‐Färbung. (a) Übersicht. (b) 100‐fache Vergrößerung. Histologisch finden sich keratinozytäre, pleomorphe Tumorzellen mit Durchbruch der Basalmembran. Zusätzlich zeigt sich ein starkes lymphozytäres Infiltrat um die Fistelgänge und das cSCC.

Obwohl die Rolle von HPV als humanes Epithelkarzinogen bei Zervix‐ und Oropharynxkarzinomen gut erforscht ist, bleibt die Rolle von HPV bei HS‐Läsionen unklar. Der Zusammenhang zwischen Rauchen und einer anogenitalen HPV‐Infektion in Verbindung mit der entzündlichen Tumormikroumgebung bei HS kann das Risiko einer Karzinogenese erhöhen.^6^

Eine Einschränkung der Fallserie ist, dass die Daten monozentrisch erhoben wurden. Das monozentrische Design der Analyse könnte zu Verzerrungen führen, da Bochum ein Referenzzentrum für schwere Fälle von HS ist.

Unsere Fälle zeigen, dass das cSCC eine schwere Komplikation der HS ist, die innerhalb kurzer Zeit zum Tod führen kann. Insgesamt treten cSCC in HS‐Läsionen bei Männern, Rauchern, Hurley‐III‐Patienten und in der Damm‐/Gesäßregion auf. Es sollte erwogen werden, HS‐Patienten mit hohem Risiko einer engmaschigen Überwachung zu unterziehen und bei Bedarf Mapping‐Biopsien durchzuführen.

## DANKSAGUNG

Open access Veröffentlichung ermöglicht und organisiert durch Projekt DEAL.

## INTERESSENKONFLIKT

N.A. erhielt Finanzmittel, Reisekostenzuschüsse und/oder persönliche Honorare für Vorträge von Novartis Pharma und Johnson & Johnson, die unabhängig von der eingereichten Arbeit waren. F.G.B. erhielt Honorare für die Teilnahme an Advisory Boards, klinischen Studien und/oder als Redner von AbbVie Inc., AbbVie Deutschland GmbH & Co. KG, Boehringer Ingelheim Pharma GmbH & Co. KG, Novartis Pharma GmbH, UCB Pharma, Incyte Corporation und Janssen Cilag GmbH, MoonLake. E.S. erhielt Vortragsvergütungen von Almirall, Leo, Pierre Fabre und Philips. L.O. erhielt Honorare als Redner und/oder Reisekostenzuschüsse von Novartis Pharma GmbH, Incyte Biosciences Corporation und Janssen. Alle übrigen Autoren (Y.H., R.K. und C.F.) erklären, dass keine Interessenkonflikte bestehen.
